# Functional Characterization of Pembrolizumab Produced in *Nicotiana benthamiana* Using a Rapid Transient Expression System

**DOI:** 10.3389/fpls.2021.736299

**Published:** 2021-09-09

**Authors:** Tanapati Phakham, Christine Joy I. Bulaon, Narach Khorattanakulchai, Balamurugan Shanmugaraj, Supranee Buranapraditkun, Chatikorn Boonkrai, Sarintip Sooksai, Nattiya Hirankarn, Yoshito Abe, Richard Strasser, Kaewta Rattanapisit, Waranyoo Phoolcharoen

**Affiliations:** ^1^Interdisciplinary Program of Biomedical Sciences, Graduate School, Chulalongkorn University, Bangkok, Thailand; ^2^Center of Excellence in Systems Biology, Faculty of Medicine, Chulalongkorn University, Bangkok, Thailand; ^3^Department of Pharmacognosy and Pharmaceutical Botany, Faculty of Pharmaceutical Sciences, Chulalongkorn University, Bangkok, Thailand; ^4^Plant-Produced Pharmaceutical Research Unit, Chulalongkorn University, Bangkok, Thailand; ^5^Baiya Phytopharm Co., Ltd., Bangkok, Thailand; ^6^Department of Microbiology, Faculty of Medicine, Center of Excellence in Immunology and Immune-Mediated Diseases, Chulalongkorn University, Bangkok, Thailand; ^7^Center of Excellence in Vaccine Research and Development (Chula Vaccine Research Center-Chula VRC), Faculty of Medicine, Chulalongkorn University, Bangkok, Thailand; ^8^The Institute of Biotechnology and Genetic Engineering, Chulalongkorn University, Bangkok, Thailand; ^9^Department of Pharmaceutical Sciences, School of Pharmacy at Fukuoka, International University of Health and Welfare, Okawa, Japan; ^10^Department of Applied Genetics and Cell Biology, University of Natural Resources and Life Sciences, Vienna, Austria

**Keywords:** *Nicotiana benthamiana*, molecular farming, transient expression, cancer immunotherapy, anti-PD-1 antibody, Pembrolizumab, plant-produced Pembrolizumab

## Abstract

The striking innovation and clinical success of immune checkpoint inhibitors (ICIs) have undoubtedly contributed to a breakthrough in cancer immunotherapy. Generally, ICIs produced in mammalian cells requires high investment, production costs, and involves time consuming procedures. Recently, the plants are considered as an emerging protein production platform due to its cost-effectiveness and rapidity for the production of recombinant biopharmaceuticals. This study explored the potential of plant-based system to produce an anti-human PD-1 monoclonal antibody (mAb), Pembrolizumab, in *Nicotiana benthamiana*. The transient expression of this mAb in wild-type *N. benthamiana* accumulated up to 344.12 ± 98.23 μg/g fresh leaf weight after 4 days of agroinfiltration. The physicochemical and functional characteristics of plant-produced Pembrolizumab were compared to mammalian cell-produced commercial Pembrolizumab (Keytruda^®^). Sodium dodecyl sulfate polyacrylamide gel electrophoresis (SDS-PAGE) and western blot analysis results demonstrated that the plant-produced Pembrolizumab has the expected molecular weight and is comparable with the Keytruda^®^. Structural characterization also confirmed that both antibodies have no protein aggregation and similar secondary and tertiary structures. Furthermore, the plant-produced Pembrolizumab displayed no differences in its binding efficacy to PD-1 protein and inhibitory activity between programmed cell death 1 (PD-1) and programmed cell death ligand 1 (PD-L1) interaction with the Keytruda^®^. *In vitro* efficacy for T cell activation demonstrated that the plant-produced Pembrolizumab could induce IL-2 and IFN-γ production. Hence, this proof-of-concept study showed that the plant-production platform can be utilized for the rapid production of functional mAbs for immunotherapy.

## Introduction

Immunotherapy is a form of cancer treatment that utilizes the immune system of a patient to target cancer cells (Pardoll, 2012). Among the types of immunotherapies, immune checkpoint inhibitors (ICIs) therapy is based on monoclonal antibody (mAb) to target immune checkpoint molecules on immune cells or cancer cells. These ICIs act by blocking and inhibiting co-stimulatory molecules between tumor cells and immune cells resulting in the enhanced T-cells activation and revival of anergic tumor-reactive T cells mounted effective antitumor responses (Marin-Acevedo et al., 2018; Wei et al., 2018). In 2018, the market value of these ICIs was more than US $34.6 billion and increasing annually (Lu et al., 2020). One of the most effective ICIs used for the cancer treatment is Pembrolizumab (Keytruda^®^) which targets human programmed cell death protein 1 (PD-1). Pembrolizumab was approved by the US Food and Drug Administration (FDA) for more than 15 cancer indications (Paul et al., 2013).

Generally, the therapeutic antibodies are mostly produced in mammalian cell cultures (Chartrain and Chu, 2008; Chames et al., 2009). The mammalian expression system is currently favorable for recombinant protein production due to the optimized manufacturing conditions and regulatory approval. However, the mammalian expression system still has some concerns, such as safety, risk of contamination, expensive raw materials, high initial investment, extensive demands, and time-consuming for the upstream process development (Li et al., 2010; Moussavou et al., 2015).

In recent days, plants are widely used for pharmaceutical and industrial protein production, such as human growth factors, cytokines, enzymes, anti-microbial peptides, vaccines, antibodies, and diagnostic reagents (Obembe et al., 2011; Donini and Marusic, 2019; Diego-Martin et al., 2020; Porngarm et al., 2020; Rattanapisit et al., 2020; Shanmugaraj et al., 2020a, 2021; Siriwattananon et al., 2020). The plant-based platforms can reduce the investment costs for upstream processing and manufacturing while addressing simple, rapid, and versatile technology for protein production in a short period of time (Buyel and Fischer, 2012; Mir-Artigues et al., 2019). Furthermore, the plants offer no limitations on scalability and flexibility because of low-cost planting and well-established transformation protocols as well as human viral safety and low risk of contamination (Buyel and Fischer, 2012; Moussavou et al., 2015; Zhang et al., 2017). Recently, *Nicotiana benthamiana* has been widely used as a model organism in basic research on the plant biology and utilized for plant molecular farming for several biopharmaceutical productions, such as mAbs (Whaley et al., 2011; Moustafa et al., 2016). The development of plant-produced mAb has achieved similar GMP requirements as those produced in mammalian cells in terms of safety, quality, lifespan, and immunogenicity (Fischer et al., 2012; Klimyuk et al., 2014; Ma et al., 2015). Therefore, the plant expression system represents a cutting-edge platform that extends potential clinical benefits for mAbs-based therapy (Fischer et al., 2012).

The present study aimed to utilize plant-based technology to produce an anti-human PD-1 antibody, Pembrolizumab, in *N. benthamiana*. The plant-produced Pembrolizumab was characterized for both physicochemical and functional properties *in vitro*. The results revealed that plant-produced Pembrolizumab displayed a similar binding affinity and PD-1/PD-L1 neutralizing activity compared with the commercial Pembrolizumab (Keytruda^®^). In addition, it stimulates T cell responses *in vitro*. Hence, this plant-produced Pembrolizumab has the potential to use as ICI for cancer immunotherapy.

## Materials and Methods

### Expression Vector Construction

The gene fragments encoding Pembrolizumab (Drug bank accession number DB09037) heavy chain (HC) and light chain (LC) were codon-optimized *in silico* using GeneArt^TM^ GeneOptimizer^TM^ software (Invitrogen, Thermo Fisher Scientific, MA, United States) for the expression in *N. benthamiana*. The plant-optimized codon sequences (as shown in Supplementary Figure 1) were synthesized (Bioneer, South Korea). Both the full-length HC and LC sequences were flanked with a murine leader sequence (Shanmugaraj et al., 2020b) at the N-terminus and a Ser-Glu-Lys-Asp-Glu-Leu (SEKDEL) sequence at the C-terminus of HC. The Pembrolizumab HC and LC constructs were double digested with *Xba*I and *Sac*I. The antibody gene fragments were purified and cloned into a geminiviral vector pBYR2eK2Md (pBYR2e) (Chen et al., 2011; Diamos and Mason, 2019). The pBYR2e-Pem-HC and pBYR2e-Pem-LC expression vectors were transformed into *Agrobacterium tumefaciens* GV3101 by electroporation. The *A. tumefaciens* cells harboring expression vectors were used for infiltration into plant leaves for recombinant antibody production.

### Plant Transformation and Protein Quantification

In this study, 6–8 weeks-old wild-type *N. benthamiana* were grown in a greenhouse under controlled conditions with 16 h light/8 h dark cycle at 28°C. *A. tumefaciens* GV3101 harboring pBYR2e-Pem-HC and pBYR2e-Pem-LC were cultivated in Luria Bertani broth supplemented with 50 mg/l kanamycin, 50 mg/l gentamicin, and 50 mg/l rifampicin at 28°C for overnight. The overnight grown *Agrobacterium* cells were used for small-scale agroinfiltration by mixing the cell suspensions at a 1:1 ratio and diluting with infiltration buffer (10 mM 2-N-morpholino-ethanesulfonic acid (MES) and 10 mM MgSO_4_, pH 5.5) to get a final OD_600_ 0.2. The plants were subjected to spot infiltration using a syringe without a needle. The infiltrated leaves were harvested on day 2, 4, 6, and 8 post-infiltration to monitor the expression of Pembrolizumab. The samples were pooled by combining three infiltrated leaf spots to reach an average of 30 mg leaf fresh weight (FW). The pooled leaf samples were extracted with 100 μl PBS buffer (137 mM NaCl, 2.7 mM KCl, 4.3 mM Na_2_HPO_4_, 1.47 mM KH_2_PO_4_, and pH 7.4) using a pestle and centrifuged at 20,000 × *g* for 5 min. The supernatant was used to quantify the plant-produced antibody by enzyme-linked immuno-absorbent assay (ELISA). Briefly, ELISA plate was coated with 50 μl of anti-human IgG-Fc fragment (ab97221, Abcam, United Kingdom) diluted (1:1,000) in PBS and incubated at 4°C overnight. The plate was washed with phosphate-buffered saline-Tween (PBST) (0.05% Tween-20 in PBS buffer) and blocked with 5% skim milk in PBS at 37°C for 2 h. Then, the plate was washed and incubated with diluted IgG1 kappa isotype antibody (ab206198, Abcam, United Kingdom) and antibody crude extracts (50 μl/well) at 37°C for 2 h. The plate was washed and incubated with 50 μl/well of HRP-conjugated anti-human kappa antibody (AP015, The Binding Site, United Kingdom) diluted (1:1,000) in PBST at 37°C for 1 h. After washing, the plate was developed using 3,3′,5,5′ tetramethylbenzidine (TMB) substrate (SurModics, MN, United States), and 50 μl/well of 1 M H_2_SO_4_ was added to stop the reaction. The absorbance was measured at 450 nm.

### Purification of Plant-Produced Pembrolizumab

About 100 g of infiltrated leaves were harvested 4 days after agroinfiltration and the leaves were homogenized with 200 ml PBS buffer. The plant crude extract was centrifuged at 26,000 × *g* at 4°C for 40 min and clarified with a 0.45-μm membrane filter. The resulting supernatant was purified by protein A affinity resin (Expedeon, United Kingdom) packed in a polypropylene column (Qiagen, Germany) with 15 mm column diameter. The proteins were washed with PBS buffer and the recombinant antibody was eluted using 0.1 M glycine at pH 2.7 and neutralized with 1.5 M Tris-HCl pH 8.8 to final pH 7.4. Purified plant-produced antibody was buffer exchanged and concentrated using Amicon^®^ Ultra (30 K) centrifugal filter (Merck, Germany) according to the instructions from the manufacturer. Purified plant-produced antibody was quantified by ELISA and used for further experiments.

### Sodium Dodecyl Sulfate Polyacrylamide Gel Electrophoresis (SDS-PAGE) and Western Blot Analysis

The sodium dodecyl sulfate polyacrylamide gel electrophoresis (SDS-PAGE) and western blot analysis of the purified plant-produced Pembrolizumab was performed under both the reducing and non-reducing conditions as described previously (Rattanapisit et al., 2019b). For the SDS-PAGE analysis, the bands (2 μg) were visualized by InstantBlue staining (Expedeon, United Kingdom). For western blot, approximately 0.05–0.5 μg of antibodies were transferred onto nitrocellulose membrane (Bio-Rad, CA, United States). The membrane was blocked with 5% skim milk in PBS and then washed with PBST. Proteins were detected either with HRP-conjugated anti-human gamma antibody (The Binding Site, United Kingdom) or HRP-conjugated anti-human kappa antibody (The Binding Site, United Kingdom) diluted (1:5,000) in PBS. The membranes were washed with PBST, developed using enhanced chemiluminescence (ECL) plus detection reagent (Abcam, United Kingdom), and recorded in a medical X-ray green (MXG) film (Carestream, GA, United States).

### Size Exclusion Chromatography

The ÄKTA Pure fast protein liquid chromatography (FPLC) purification system was used to assess protein purity and protein aggregations. In brief, antibody samples (0.5 mg/ml, 100 μl) were injected into the Superdex^®^ 200 Increase 10/300 GL column (Cytiva, MA, United States). A PBS buffer was used as a running buffer with a flow rate of 0.5 ml/min. The absorbance monitored the chromatogram of each antibody sampled at 280 nm.

### Circular Dichroism (CD) Spectroscopy

Before measurements, the plant-produced and commercial Pembrolizumab antibodies were concentrated by Amicon^®^ Ultra (30 K) (Merck, Germany). Their concentrations were determined using their extinction coefficients at 280 nm that were calculated by their amino acid sequence. Then, their concentrations were adjusted to 10 μM using PBS buffer at pH 7.4. The CD spectra were recorded at room temperature using a quartz cell with a 1-mm optical path length on a J-720W CD spectropolarimeter (JASCO, Japan). The molar ellipticity expressed in degrees × cm^2^/dmol was calculated based on a mean residue molecular weight of 110.

### NMR Spectroscopy

NMR spectra were recorded on a Varian Unity INOVA 600 spectrometer (Varian, CA, United States). For NMR measurements, the antibody concentrations were adjusted to 100 μM using PBS buffer at pH 7.4 containing 10% v/v D_2_O. Topspin 4.1.1 software (Bruker Corporation, MA, United States) was used to process the data.

### N-Glycan Analysis

Purified plant-produced antibody was subjected to SDS-PAGE under non-reducing conditions. The target protein band was excised from the gel, S-alkylated, and digested with trypsin. Liquid chromatography-electrospray ionization-mass spectrometry (LC-ESI-MS) of tryptic glycopeptides was performed as described previously (Strasser et al., 2008).

### PD-1 Binding Profile by ELISA

The PD-1 binding activity of plant-produced Pembrolizumab was determined by ELISA. Briefly, the ELISA plate was coated with 100 μl/well of recombinant human PD-1 His tag protein at 0.1 μg/ml (8986-PD, R&D Systems, MN, United States) at 4°C for overnight. The plate was washed and blocked with PBST. The two-fold serial dilutions of anti-PD-1 antibodies or human IgG4 isotype control (403701, BioLegend, CA, United States) starting from 2 μg/ml (100 μl/well) were added and the plate was incubated at 37°C for 1 h. Then, the HRP-conjugated goat anti-human IgG antibody (109035088, Jackson ImmunoResearch, PA, United States) diluted (1:10,000) in PBST was added and incubated at 37°C for 1 h. The plate was washed and developed with 100 μl/well of SigmaFast^TM^ OPD substrate solution in the dark at room temperature for 20 min. The reaction was stopped by adding 50 μl/well of 1 M H_2_SO_4_ and the absorbance was measured at 492 nm using a Cytation^TM^ five cell imaging multi-mode reader.

### PD-1 Binding Kinetics by Surface Plasmon Resonance (SPR)

The Biacore T200 equipped with a protein G sensor chip (chip ID. 10258853, GE Healthcare, IL, United States) was used to determine the binding kinetics of anti-PD-1 antibodies. In the protein-capturing step, anti-PD-1 antibodies in HBS-EP running buffer at 3 μg/ml were injected into an individual flow cell of the protein G sensor chip. A single-cycle kinetic was performed to determine the binding kinetics by injecting five different concentrations (10, 20, 40, 80, and 160 nM) of human PD-1 His tag (8986-PD, R&D Systems, MN, United States) at a flow rate of 30 μl/min with association time for 60 s and dissociation time for 120 s. The signal of an uncoated reference cell was subtracted from the sensor grams, and the HBS-EP buffer blank was also included as a negative control for double referencing. The Biacore T200 evaluation software version 3.1 was used for the calculation of association rate constant (*k*_*on*_), dissociation rate constant (*k*_*off*_), and equilibrium dissociation constant (*K*_*D*_) by curve fitting the data with a Langmuir 1:1 binding model.

### PD-1/PD-L1 Blockade Assay

The PD-1/PD-L1 neutralizing activity was determined by cell-based luciferase reporter assay (PD-1/PD-L1 blockade bioassays, Promega, WI, United States). Briefly, PD-L1 aAPC/CHO-K1 cells were seeded into a white flat-bottom 96-well plate and incubated in a 5% CO_2_ humidified incubator at 37°C for 16 h. Three-fold serial dilutions of anti-PD-1 antibodies and the PD-1 effector cells were added to the plate and incubated in 5% CO_2_ humidified incubator at 37°C for 6 h. After co-culture, the Bio-Glo^TM^ substrate reagent was added to the plate and incubated at room temperature for 5 min. The luminescence signal was measured using a Cytation^TM^ 5 cell imaging multi-mode reader and reported as relative light units (RLUs).

### Production of Cytokines

The peripheral blood mononuclear cells (PBMCs) were separated from healthy blood donors by density gradient centrifugation with Isoprep (Robbins Scientific Corporation, CA, United States). The isolated PBMCs were resuspended in fetal bovine serum (FBS) with 10% dimethyl sulfoxide (DMSO) and kept frozen until assay time. On day 0, frozen PBMCs (*n* = 4) were thawed and seeded at 1 × 10^5^ cells/well in the assay plate. The cells were stimulated with the Staphylococcal enterotoxin B (SEB) (Rattanapisit et al., 2019b) at 1 ng/ml in the presence of antibodies at 0.01 and 0.1 μg/ml. Keytruda^®^ and human IgG4 antibody (BioLegend, CA, United States) were used as positive and negative control, respectively. On day 3, the secretion levels of IL-2 and IFN-γ in culture supernatant were determined by ELISA (BioLegend).

### Statistical Analysis

All the experiments in the study were performed three times. A statistical analysis was performed using GraphPad Prism 8.0 (GraphPad Software, CA, United States). A multiple *t*-test was utilized to determine statistically significant differences between each group using the Holm–Sidak method, with alpha equal to 0.05. A *P* value less than 0.05 (*P* ≤ 0.05) was considered as statistically significant.

## Results

### Rapid Transient Expression of Pembrolizumab *in N. benthamiana*

**FIGURE 1:**
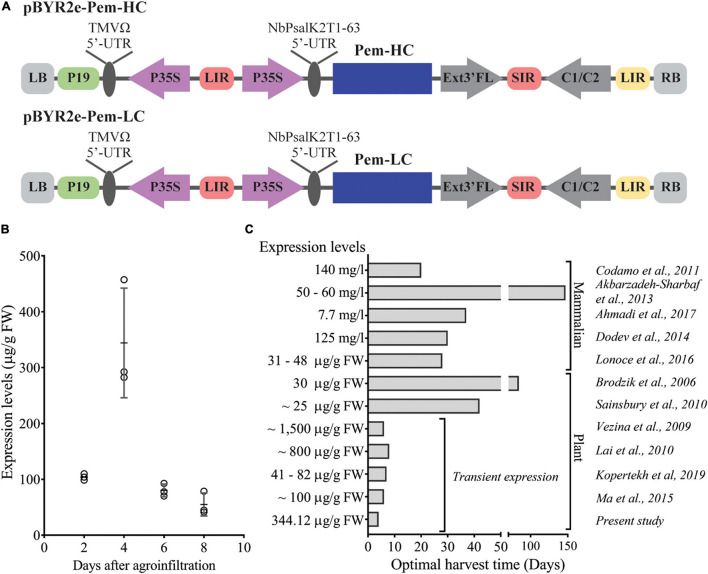
Rapid transient expression of Pembrolizumab in *N. benthamiana*. **(A)** Schematic representation of the T-DNA regions of the expression vectors used for the transient expression of Pembrolizumab in *N. benthamiana*. P19: the RNA silencing suppressor gene derived from tobacco bushy stunt virus (TBSV); P35S: Cauliflower Mosaic Virus (CaMV) 35S promoter; TMVΩ 5′-UTR: 5′ untranslated region (UTR) of tobacco mosaic virus Ω; NbPsalK2T1-63 5′UTR: 5′ UTR of *Nicotiana* photosystem I reaction center subunit *psaK*; Pem-HC: heavy chain of Pembrolizumab; Pem-LC: light chain of Pembrolizumab; Ext3′FL: 3′ region of tobacco extension gene; C1/C2: Bean Yellow Dwarf Virus (BeYDV) ORFs C1 and C2 encoding for replication initiation protein (Rep) and RepA; SIR and LIR: short and long intergenic regions of the BeYDV genome; LB and RB: left and right borders of the *Agrobacterium* T-DNA region. **(B)** The antibody expression levels in wild-type *N. benthamiana* on day 2, 4, 6, and 8 after agroinfiltration were quantified by ELISA. Data are presented as mean ± SD. **(C)** Comparison between the optimal harvest time and the recombinant antibody expression levels with previous reports. Cost comparison of these mAbs production in plants and mammalian system was not performed.

To evaluate the expression of Pembrolizumab, *Agrobacterium* harboring the heavy chain (Pem-HC) and light chain (Pem-LC) expression vectors (Figure 1A) were co-infiltrated into the plant leaves. An 8-day expression time-course experiment was performed and infiltrated leaves were harvested every 2 days after agroinfiltration. The expression levels of Pembrolizumab were quantified by ELISA and were reported as microgram per gram (μg/g) leaf FW. The results showed that the maximum expression level of antibody was observed 4 days after agroinfiltration reaching up to 344.12 ± 98.23 μg/g FW as shown in Figure 1B and Supplementary Table 1. However, the expression level of Pembrolizumab was 4-fold declined from day 6 to 8 post-infiltration. All infiltrated leaf samples that showed a significant reduction in antibody expression also displayed rapid wilting and apparent necrosis at the infiltrated site (data not shown), potentially affecting the antibody levels obtained from the leaves harvested on day 6 and 8. We also demonstrated that this rapid transient expression platform could be used for recombinant antibody production within 4 days after infiltration when compared with other expression platforms, as indicated in Figure 1C and Supplementary Table 2.

### Physicochemical Characteristics of Purified Plant-Produced Pembrolizumab

**FIGURE 2:**
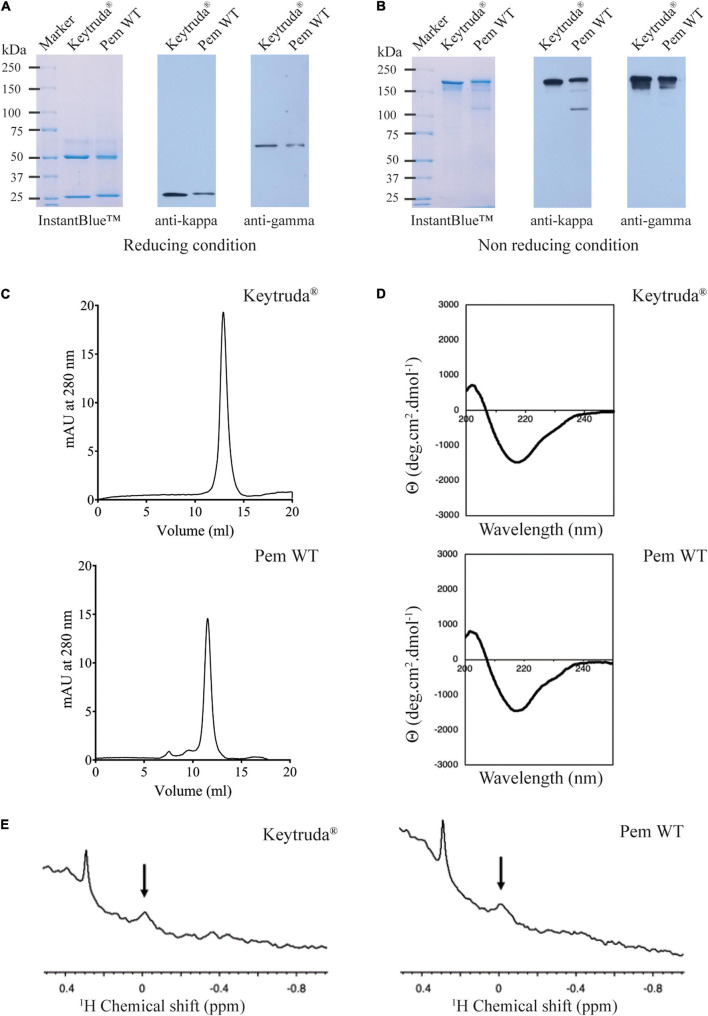
Physicochemical characteristics of purified plant-produced Pembrolizumab. Sodium dodecyl sulfate polyacrylamide gel electrophoresis (SDS-PAGE) (4–15%) and western blot analysis of plant-produced Pembrolizumab (Pem WT) under **(A)** reducing condition and **(B)** non-reducing condition. For the SDS-PAGE, proteins were stained with InstantBlue^TM^ staining solution. For the western blot analysis, proteins were probed with either HRP-conjugated anti-human kappa or anti-human gamma and detected with enhanced chemiluminescence (ECL) substrate solution. **(C)** Chromatogram of Keytruda^®^ (top) and Pem WT (bottom) analyzed by size-exclusion chromatography. **(D)** Circular dichroism spectrum of Keytruda^®^ (top) and Pem WT (bottom) in PBS at pH 7.4. **(E)** NMR spectra in methyl region of Keytruda^®^ (left) and Pem WT (right) in PBS buffer at pH 7.4 containing 10% v/v D_2_O at 25°C.

As we have successfully obtained plant-produced Pembrolizumab, the characterization of its molecular features was subsequently performed. A gradient 4–15% SDS-PAGE (reducing and non-reducing conditions) and western blot analysis were performed with plant-produced Pembrolizumab. Under the reducing condition, HC and LC were observed at approximately 50 and 25 kDa, respectively. A western blot analysis with anti-human kappa antibody and anti-human gamma antibody also confirmed the expression of both HC and LC with the expected molecular size comparable to Keytruda^®^ (Figure 2A). Meanwhile, the results under the non-reducing condition revealed that plant-produced antibody exhibited assembly into its tetrameric form, which was found at 150 kDa, as shown in Figure 2B. However, minor amounts of antibody fragments were also observed, as indicated by the additional bands in the SDS-PAGE gels.

Size exclusion chromatography was performed to assess the antibody aggregation. The results demonstrated that plant-produced Pembrolizumab assembled as full IgG molecule (major peak) as shown in Figure 2C with a relatively low-level of antibody aggregates (small minor peaks). No fragmented forms of the antibodies were observed in the chromatogram. Furthermore, the secondary structure of plant-produced Pembrolizumab was compared with Keytruda^®^ using CD spectroscopy. The CD results demonstrated that the secondary structure of plant-produced Pembrolizumab was comparable to that of the Keytruda^®^ (Figure 2D). Both the spectra denote negative absorbance at 218 nm, which infers a β-sheet-rich structure. Further, the tertiary structure of plant-produced Pembrolizumab was compared with Keytruda^®^ using NMR spectroscopy. The up-fielded methyl protons were observed in both spectra, indicating that the tertiary structures are retained (Figure 2E). The peaks at the same chemical shifts in NMR spectra also confirmed that the tertiary structures are similar for plant-produced Pembrolizumab and Keytruda^®^.

### N-Glycosylation Profile

**FIGURE 3:**
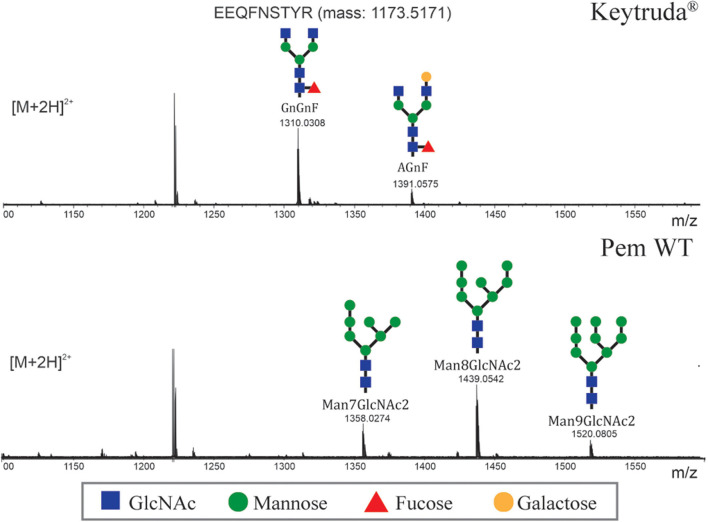
N-glycosylation profile of plant-produced Pembrolizumab. The N-glycosylation profile of the glycopeptide EEQFNSTYR is shown, and the major glycosylated peaks [M + 2H]^2+^ are depicted. For a detailed explanation of glycan structure abbreviations, see www.proglycan.com.

Generally, the early stages of N-glycosylation are similar between plant and mammalian cells whereas the maturation steps responsible for the complex glycan differs between these systems. To evaluate the *N*-glycan profile of plant-produced Pembrolizumab, LC-ESI-MS was used (Figure 3). Based on the results, the *N*-glycan profile of Keytruda^®^ revealed the presence of mammalian-type *N*-glycan species, such as GlcNAc2Man3FucGlcNAc2 (GnGnF) and GalGlcNAc2Man3FucGlcNAc2 (AGnF) as expected for a mammalian-cell produced mAb. By contrast, the plant-produced Pembrolizumab displayed oligomannosidic *N*-glycans, i.e., Man7GlcNAc2, Man8GlcNAc2, and Man9GlcNAc2 which are typical for endoplasmic reticulum (ER)-retained glycoproteins. However, the *N*-glycans in plant-produced Pembrolizumab did not affect the binding properties or binding affinity of the antibody with its target.

### Functional Characterization of Plant-Produced Pembrolizumab

It is known that plants have different molecular machinery for protein production and post-translational modifications in comparison with the mammalian system (Gomord and Faye, 2004). Thus, we focused on the analysis of functional characteristics of plant-produced Pembrolizumab.

**FIGURE 4:**
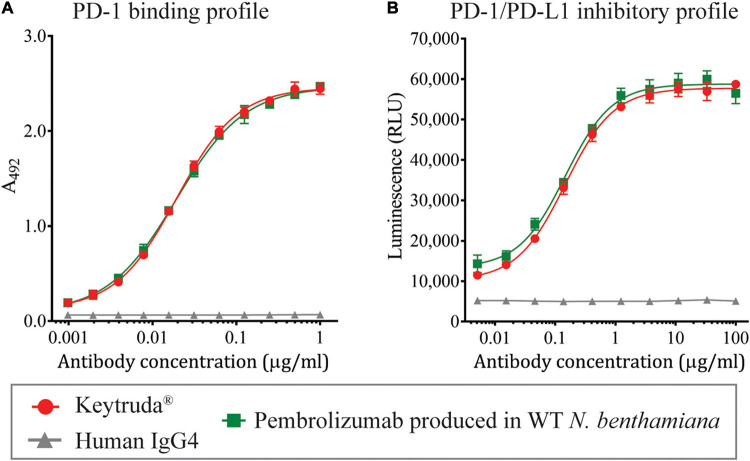
Binding and inhibition profiles of plant-produced Pembrolizumab. Purified plant-produced Pembrolizumab was assessed for the **(A)** binding activity with human PD-1 by ELISA and **(B)** PD-1/PD-L1 inhibition activity by PD-1/PD-L1 blockade bioassay. The Keytruda^®^ and human IgG4 were used as positive and negative control, respectively. Data are presented as mean ± SD.

To determine the functional characteristics of plant-produced Pembrolizumab, ELISA was performed to evaluate the binding activity to human PD-1, while the PD-1/PD-L1 cell-based blockade bioassay was used to assess the inhibitory action. Serial dilutions of each antibody sample were added to the PD-1-coated ELISA plate, and the goat anti-human IgG-HRP antibody was used to detect the antibody that was specifically bound to the PD-1 protein. The specific binding results demonstrated that the plant-produced Pembrolizumab showed similar dose-dependent binding activity with human PD-1 protein compared with Keytruda^®^ (Figure 4A), while the negative control human IgG4 antibody did not exhibit any binding to human PD-1 protein. Furthermore, the inhibitory activity of plant-produced Pembrolizumab was assessed. Serial dilutions of each antibody sample and Jurkat/PD-1 effector cell were added to the assay plate containing pre-cultured CHO-K1/PD-L1 cells, and the plate was incubated for 6 h. The presence of anti-human PD-1 antibody inhibits the interaction between PD-1 and PD-L1 resulting in the activation of luciferase reporter gene. The results indicated that plant-produced Pembrolizumab inhibited the interaction between PD-1 and PD-L1 in a dose-dependent manner (Figure 4B) with a half-maximal effective concentration (EC_50_) of 147.2 ng/ml compared with Keytruda^®^ (EC_50_ = 146.7 ng/ml). These results confirmed that the plant-produced Pembrolizumab displayed functional binding to human PD-1 and inhibits PD-1 and PD-L1 interaction *in vitro* at a comparable level with Keytruda^®^.

**FIGURE 5:**
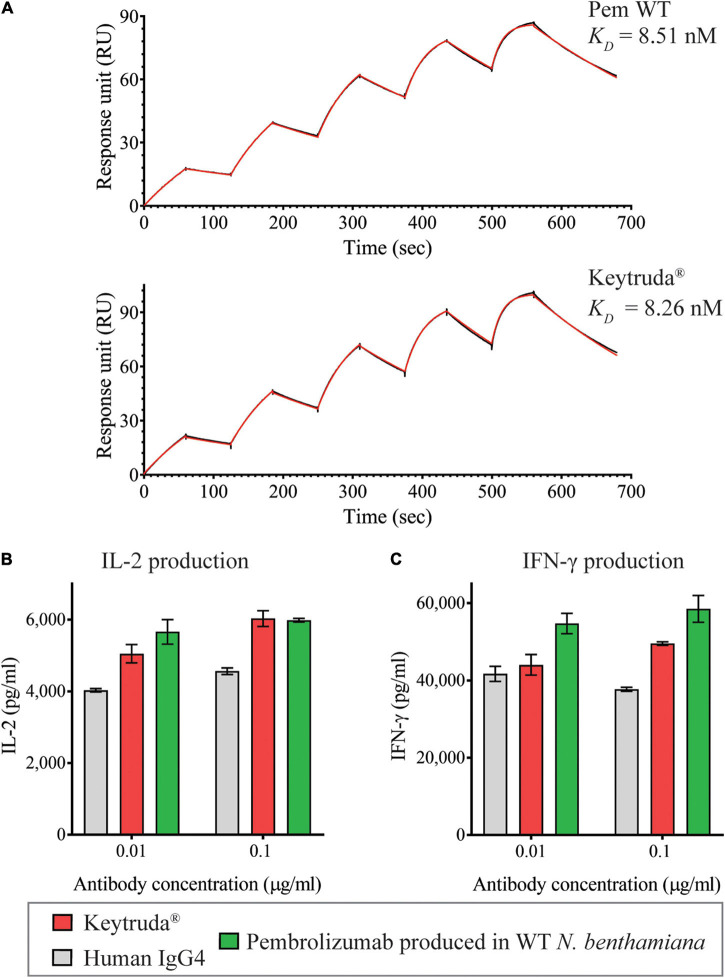
Functional characterization of plant-produced Pembrolizumab. **(A)** Binding kinetics data of plant-produced Pembrolizumab and Keytruda^®^ with human PD-1 analyzed by SPR. PBMCs from healthy donors were stimulated with 1 ng/ml SEB in the presence of antibodies at 0.01 and 0.1 μg/ml. The secretion levels of **(B)** IL-2 and **(C)** IFN-γ in culture supernatant after co-culture for 3 days were measured by ELISA. Data are presented as mean ± SD.

Since plant-produced Pembrolizumab has exhibited both binding and inhibitory functions, we then, determined the binding kinetics of plant-produced Pembrolizumab with human PD-1 protein. A single-cycle binding kinetics was performed using surface plasmon resonance (SPR). Data showed that the equilibrium dissociation constant (*K*_*D*_) of plant-produced Pembrolizumab was 8.51 nM, while the *K*_*D*_ of Keytruda^®^ was 8.26 nM, as shown in Figure 5A and Supplementary Table 3. Both antibodies exhibited subnanomolar binding affinity with its target. These data confirmed that the plant-produced Pembrolizumab has high binding affinity with human PD-1 protein.

This study successfully confirmed that plant-produced Pembrolizumab has binding and inhibition activities with its target. So, we further examined the potency of the antibody on T cell activation *in vitro*. The SEB stimulation of PBMCs in the presence of either anti-PD-1 antibodies or human IgG4 antibody control at 0.01 and 0.1 μg/ml was performed. Antigen-presenting cell and effector T cells were co-cultured in the presence of SEB toxin. The SEB toxin stimulates T cell response *in vitro*. However, when T cells are activated for a while, regulatory mechanisms break the over-induction signals in PD-1/PD-L1 pathway. The presence of anti-PD-1 antibody inhibits the regulatory mechanism. Hence, T cell becomes activated and secrets activating cytokines, such as IFN-gamma and IL-2. On the other hand, without anti-PD-1 antibody in the assay plate, antigen-presenting cell and effector T cells maintain a balance between stimulatory and regulatory mechanism. The results showed that the plant-produced Pembrolizumab and Keytruda^®^ (at 0.1 μg/ml) were significantly able to stimulate T cell responses *via* IL-2 (Figure 5B) and IFN-γ (Figure 5C) secretion compared with the human IgG4 antibody control. Noticeably, plant-produced Pembrolizumab even at a low dose (0.01 μg/ml) significantly induced both IL-2 and IFN-γ production. At the same time, Keytruda^®^ displayed no significant in IFN-γ production compared with human IgG4 control. These findings revealed that plant-produced Pembrolizumab promoted T cells responses *in vitro*.

## Discussion

Immunotherapy represents an innovative approach for the treatment of multiple types of cancer. Several immune checkpoint molecules have been discovered and explored over the years, such as programmed cell death 1 (PD-1), programmed cell death ligand 1 (PD-L1), and cytotoxic T-lymphocyte antigen 4 (CTLA-4) (He and Xu, 2020). The development of ICIs against these inhibitory immunoreceptors has potential benefits and shaped the therapeutic ways of several cancer types. Ever since, these drugs have translated to a great deal of success in cancer immunotherapy (Robert et al., 2014; Weber et al., 2015; Kasamon et al., 2017; Liu and Cho, 2017).

Generally, ICIs used for cancer treatment were produced in mammalian cells. However, the investment costs and production process of ICIs produced from mammalian-based are varied and took longer time. Because the investment cost and time for phase 1 cGMP manufacturing of plant expression system are comparatively cost-effective (7.5- to 10-fold) and faster (<6 months) than mammalian expression system, the plant-production platform might be an alternative platform for the production of many biopharmaceutical products. The use of plant expression systems for the production of pharmaceutically important proteins (Bulaon et al., 2020; Hanittinan et al., 2020), vaccines (Marsian et al., 2017; Rosales-Mendoza et al., 2017), diagnostic reagents (Rattanapisit et al., 2021), and antibodies (Kopertekh et al., 2019; Hurtado et al., 2020; Rattanapisit et al., 2020) were documented. In addition, the prior studies reported that the generation of plant-made protective immunogen and therapeutic antibodies (Dent et al., 2016; Rattanapisit et al., 2019a,b; Nessa et al., 2020; Yiemchavee et al., 2021).

Herein, we successfully produced Pembrolizumab using a rapid transient expression system in *N. benthamiana*. This study utilized the benefits of plant viral vectors in terms of speed and yield to produce a recombinant anti-human PD-1 antibody for effective cancer immunotherapy. In particular, a geminiviral vector pBYR2e based on the bean yellow dwarf virus (BeYDV) was used (Diamos and Mason, 2019). It contains the self-rolling circle replication elements, which can produce a high copy number of the expression cassette eventually resulting in a high accumulation of recombinant proteins in plants (Huang et al., 2010; Chen, 2018). More so, the use of viral vectors for transient expression in *N. benthamiana* has proven its efficiency as a suitable host for viral infectivity (Goodin et al., 2008). In particular, mAbs against enterovirus infection (Rattanapisit et al., 2019a), porcine epidemic diarrhea virus infection (Rattanapisit et al., 2017), and even the recent coronavirus infection (Shanmugaraj et al., 2020b) were transiently expressed in *N. benthamiana* using this geminiviral vector. The optimal yields obtained vary from 4 to 130 μg/g leaf fresh weight within 3–6 days after infiltration. Due to these advantages, geminiviral vectors have been utilized for transient production of therapeutically important mAbs in plants. Intriguingly, other studies utilized the plant expression system for the production of anti-human immunodeficiency virus (HIV) mAb (Sainsbury et al., 2010), tumor-targeting mAb (Vaquero et al., 1999; Villani et al., 2009), HCG-specific mAb (Kathuria et al., 2002), murine anti-human IgG C5-1 (Vézina et al., 2009), anti-West Nile virus mAb (Lai et al., 2010), and reported varying levels of antibody accumulation.

The SEKDEL motif at the C-terminus of the HC was added for ER retention to improve protein accumulation in the ER (Petruccelli et al., 2006). The folding and assembly of newly synthesized proteins to form mature complex protein in the ER begins prior complete translation of polypeptide (Farràs et al., 2020) and final transport to cellular destination (Yamamoto et al., 2001). In this work, we adapted this principle and hypothesize that SEKDEL-tagged HC could stabilize initially by forming disulfide-bonded dimers, to which LC can be assembled by forming disulfide bond between constant domains (C_*L*_ and C_*H*_1) (Feige et al., 2010; Weiner, 2015), eventually the fully assembled antibody can be retrieved from cis-Golgi back to the ER for retention and subsequent protein accumulation.

The results demonstrated that Pembrolizumab was expressed rapidly in *N. benthamiana* at the highest level of expression obtained within 4 days post-infiltration providing essential advantages of speed over transgenic plant expression system (Brodzik et al., 2006; Ma et al., 2015; Lonoce et al., 2016), mammalian expression system (Codamo et al., 2011; Akbarzadeh-Sharbaf et al., 2013; Dodev et al., 2014; Ahmadi et al., 2017), and in some transient expression systems (Sainsbury et al., 2010; Kopertekh et al., 2019). The maximum expression level of Pembrolizumab reached up to 344.12 ± 98.23 μg/g FW after 4 days of post-infection followed by a marked decrease after 6 days. The significant drop in antibody expression might be due to the progressive development of necrosis on the infiltrated leaves observed from day 6 to 8 post-infiltration. The high-level of necrosis, eventually resulting in cell death, is considered a critical factor for reduced protein yield (Mathew et al., 2014; Hamorsky et al., 2015).

The results from size-exclusion chromatography revealed that plant-produced Pembrolizumab efficiently assembled into the whole IgG molecule and displayed the tetrameric isoform. The protein impurities and IgG aggregates were observed at small amounts, supporting the intrinsic aggregation propensity of all therapeutic proteins, such as antibodies (Roberts, 2014). Aggregation is considered either process or product-related impurities that must be monitored and controlled to a minimum extent (Cleland et al., 1993). Moreover, antibody fragments were not observed in the size exclusion chromatogram prospectively due to the low concentration of fragments in the sample solution (Adawy and Groves, 2017). Likewise, the results from CD confirmed similar secondary structures of the Pembrolizumab produced in a plant and a mammalian cell. This study findings from NMR spectroscopy also presented similarities on the tertiary structures of the plant-derived Pembrolizumab and mammalian cell-derived Pembrolizumab.

The earlier stages of *N*-glycan processing in the ER are highly conserved across the species but differ significantly during the late stages in the Golgi apparatus. In particular, the plant-produced recombinant proteins contain plant-specific glycans, such as β1,2-xylose and core α1,3-fucose (Strasser, 2016), which are of concern for human applications. Nonetheless, no severe allergic reactions or hypersensitive indications were previously documented from such plant-specific *N*-glycans (Ma et al., 2015; Pillet et al., 2019). The glycan profile data confirmed that the plant-produced Pembrolizumab displayed oligomannosidic *N*-glycans attributed to targeted retention in the ER due to SEKDEL sequence. Similar to the prior reports, SEKDEL-tagged antibodies displayed non-immunogenic high-mannose *N*-glycans (Sriraman et al., 2004). However, high-mannose *N*-glycans in the plant-produced antibodies contributes for increase in the antibody clearance rate from circulation (Reusch and Tejada, 2015). Hence, glycan-engineered plants could be used instead to obtain more mammalian-like *N*-glycans that have more potentially favorable properties for therapeutic applications.

Furthermore, the functional characteristics of plant-produced Pembrolizumab were evaluated. The binding activity and binding kinetics data confirmed that the mAb effectively binds to human PD-1 protein with a high affinity similar to Keytruda^®^. It also inhibits the interaction between PD-1 and PD-L1 with equivalent EC_50_ values compared to the commercial mAb. The different RU in SPR was noted between both the antibodies which might be due to the difference in the antibody concentration captured on a protein G sensor chip. The results of the SEB stimulated PBMCs confirmed that the plant-produced Pembrolizumab could induce and promote T cells responses *in vitro*. These findings are similar to the previous report (Rattanapisit et al., 2019b).

## Conclusion

We have demonstrated the feasibility of rapid transient expression of Pembrolizumab in *N. benthamiana.* The plant-produced Pembrolizumab has *in vitro* physicochemical and functional characteristics quite similar to mammalian cell-produced Pembrolizumab. Future studies will focus on analyzing the *in vivo* efficacy of plant-produced Pembrolizumab in animal models. Altogether, this proof-of-concept study proved the robustness of the plant expression system for the production of anti-PD-1 Pembrolizumab, which could be used as employed for cancer immunotherapy.

## Data Availability Statement

The original contributions presented in the study are included in the article/Supplementary Material, further inquiries can be directed to the corresponding author/s.

## Author Contributions

KR and WP designed all the experiments. CJIB, NK, BS, and KR performed antibody gene synthesis, antibody expression, purification, and quantification. YA performed structural characterization. RS performed *N*-glycan analysis. TP, SB, CB, SS, and NH performed the binding, binding kinetics, blockade bioassay, and *in vitro* functional assays. All authors analyzed the data and contributed to manuscript preparation.

## Conflict of Interest

KR and BS are employed by Baiya Phytopharm Co., Ltd. WP is a co-founder/shareholder of Baiya Phytopharm Co., Ltd. WP is a co-founder/shareholder of Baiya Phytopharm Co., Ltd. The remaining authors declare that the research was conducted in the absence of any commercial or financial relationships that could be construed as a potential conflict of interest.

## Publisher’s Note

All claims expressed in this article are solely those of the authors and do not necessarily represent those of their affiliated organizations, or those of the publisher, the editors and the reviewers. Any product that may be evaluated in this article, or claim that may be made by its manufacturer, is not guaranteed or endorsed by the publisher.
