# Supplementary material for: Functional Characterization of Pembrolizumab Produced in Nicotiana benthamiana Using a Rapid Transient Expression System
Source: Front Plant Sci. 2021 Sep 9;12:736299. doi: 10.3389/fpls.2021.736299 (PMC8459022; doi:10.3389/fpls.2021.736299)
Supplement: Supplementary file 1 [file Data_Sheet_1.docx]

Functional characterization of Pembrolizumab produced in *Nicotiana benthamiana* using a rapid transient expression system

Tanapati Phakham^1,2^, Christine Joy I. Bulaon^3,4^, Narach Khorattanakulchai^3,4^, Balamurugan Shanmugaraj^5^, Supranee Buranapraditkun^6,7^, Chatikorn Boonkrai^1,2^, Sarintip Sooksai^8^, Nattiya Hirankarn^6^, Yoshito Abe^9^, Richard Strasser^10^, Kaewta Rattanapisit^5*^, Waranyoo Phoolcharoen^3,4*^

Supplementary Material

#
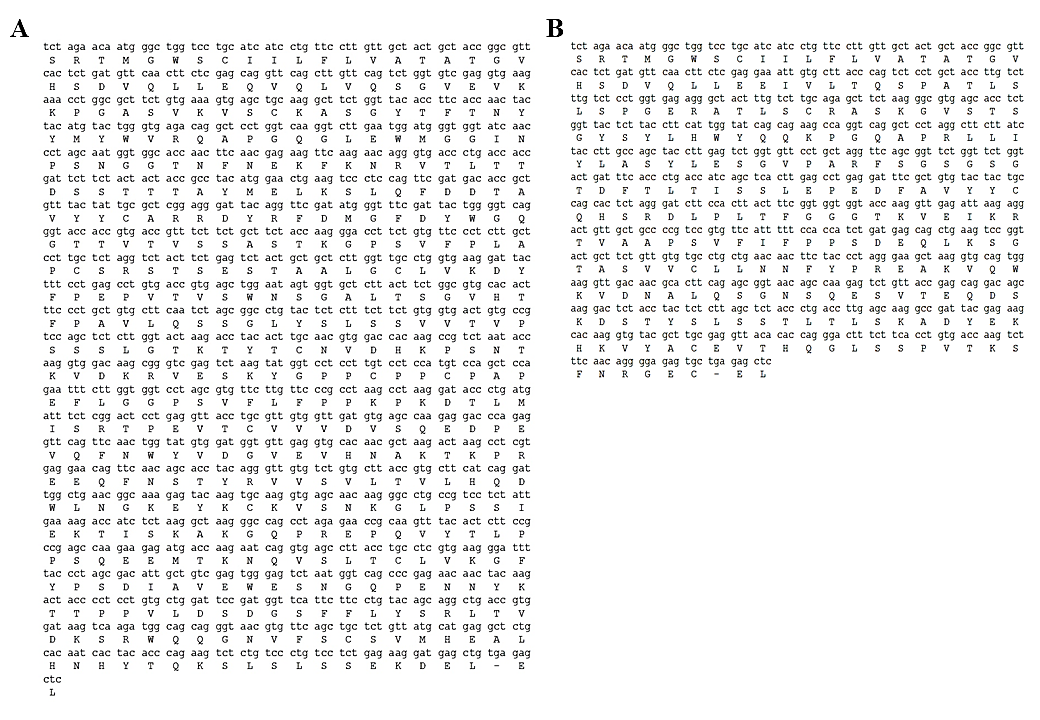
Supplementary Figures

**Supplementary Figure 1.** The nucleotide and amino acid sequences of codon-optimized Pembrolizumab (A) heavy chain and (B) light chain.

# Supplementary Table

# Supplementary Table 1: The expression levels of antibody in *N. benthamiana* on 2, 4, 6, and 8 days after agroinfiltration

| Day of harvest | Pembrolizumab expression (µg/g FW) | | | Average  (µg/g FW) |
| --- | --- | --- | --- | --- |
|  | #1 | #2 | #3 |  |
| D2 | 110.01 | 103.98 | 98.46 | 104.15 ± 5.78 |
| D4 | 292.52 | 457.40 | 282.45 | 344.12 ± 98.23 |
| D6 | 92.68 | 77.82 | 70.09 | 80.20 ± 11.49 |
| D8 | 41.39 | 44.79 | 78.66 | 54.94 ± 20.60 |

Expression levels of Pembrolizumab measured by ELISA and reported as µg/g leaf fresh weight (FW). Data were calculated from three leaf samples (#1, #2, #3) that were harvested on day 2, 4, 6, and 8 post-infiltration (D2, D4, D6, D8) and pooled for analysis.

# Supplementary Table 2: Comparison of different recombinant antibody production platforms

| Host | Expression system | Vector | Culture conditions | Expression Level | Times | Ref. |
| --- | --- | --- | --- | --- | --- | --- |
| *Nicotiana benthamiana* | Transient – agroinfiltration  [Lab scale] | pBYR2e | Greenhouse - with 16 h light/8 h dark cycle at 28°C | 344.12 ± 98.23 µg/g FW | 4 days | Present  study |
| *Nicotiana benthamiana* | Transient – agroinfiltration  [Lab scale] | pM81-FSC1 and pBINPLUS | Glasshouses maintained at 23–25°C with a 16-hour photoperiod | ~ 100 µg/g FW | 6 days | Sainsbury, et al., 2010 |
| *Nicotiana benthamiana* | Transient – agroinfiltration  [Lab scale] | pLH-γb | n/a | 41 – 82.5 µg/g FW | 6 days | Kopertekh, et al, 2019 |
| *Nicotiana benthamiana* | Transient – agroinfiltration | pICH21595 and pICH11599 | Greenhouse with a 16/8 hr light/dark cycle at 25 °C | 800 µg/g  FW | 8 days | Lai et al., 2010 |
| *Nicotiana benthamiana* | Transient – agroinfiltration | pCAMBIA with coexpression of the HcPro | glasshouse under a 16-h/8-h photoperiod with 25 °C day/20 °C night | 1,500 µg/g  FW | 6 days | Vezina et al., 2009 |
| *Nicotiana tabacum* | Stable - transgenic plants  [Manufacturing scale] | pTRAp | Greenhouse with 16 h light/8 h dark cycle at 25/22°C day/night | ~25 µg/g FW in T3 plants | 6 weeks after sowing | Ma, et al., 2015 |
| *Nicotiana tabacum* | Stable - transgenic plants  [Lab scale] | pBI121 | Greenhouse | 30 mg/kg FW in T1 plants | 10 – 12 weeks | Brodzik, et al., 2006 |
| Hairy root cultures of *N. tabacum* and ΔXTFT *N. benthamiana* | Hairy root culture from transgenic plants expressing mAb  [Lab scale] | n/a | Shake flask – 40 mL MS medium with shaking (80 rpm) at 26°C. | 31.3 – 48.7 µg/g FW | 25 – 28 days post-inoculation | Lonoce, et al., 2016 |
| HEK 293-F | Stable pool expression  [Lab scale] | pVITRO1 | 5L Wave bioreactor | 125 mg/l | 30 days | Dodev, et al., 2014 |
| CHO-S | Stable pool expression  [Lab scale] | pB513B1 and pB200A | Shake flask – 100 ml culture volume | 7.7 mg/l | 37 days | Ahmadi, et al., 2017 |
| CHO DG44 | Stable expression  [Lab scale] | pcDNA™ 3.3 for HC  pOptiVEC™ for LC | Shake flask – 30 ml culture volume | 50 – 60 mg/l | > 21 weeks | Akbarzadeh-Sharbaf, et al., 2013 |
| CHO-T and CHO-K1 | Transient expression  [Lab scale] | pPyEBV | Shake flask – 50 ml culture | 140 mg/l | 20 days | Codamo, et al., 2011 |

**Binding kinetics analysis by SPR**

# Supplementary Table 3: Binding kinetics of plant-produced Pembrolizumab (Pem WT) and commercial Pembrolizumab (Keytruda^®^) with human PD-1

| Sample | *k_on_* (1/Ms) | *k_off_* (1/s) | *K_D_* (nM) |
| --- | --- | --- | --- |
| Pem WT | 3.939×10^5^ | 3.352×10^-3^ | 8.51 |
| Keytruda^®^ | 4.1032×10^5^ | 3.389×10^-3^ | 8.26 |
